# Feasibility analysis of automated cleaning in biopharmaceutical production using cleaning-in-place concepts from food production

**DOI:** 10.3389/fmedt.2025.1540779

**Published:** 2025-09-22

**Authors:** Ferdinand Groten, Chris Henze, Matthias Joppa, Laura Herbst, Bastian Nießing, Marc Mauermann, Robert H. Schmitt

**Affiliations:** ^1^ Department for Bio-Adaptive Production, Fraunhofer Institute for Production Technology IPT, Aachen, Germany; ^2^ Fraunhofer-Institute for Process Engineering and Packaging IVV, Dresden, Germany; ^3^ Laboratory for Machine Tools and Production Engineering (WZL), RWTH Aachen University, Aachen, Germany

**Keywords:** automation, decontamination, cleaning, CIP, biopharmaceutical industry, food production

## Abstract

In biopharmaceutical production involving cells, cell-derived products, or tissues, the cleaning of surfaces that come into direct or indirect contact with the product is currently performed mostly by hand as the initial step in decontamination. This manual approach leads to production inefficiencies, reduced reproducibility of decontamination processes, and product losses. In food production, automated processes are preferred for the decontamination of interior product contact surfaces. This article studies the feasibility of adapting the automated cleaning-in-place concepts used in the food industry to biopharmaceutical production. The focus is on spray cleaning processes and validation by cleaning simulation. An existing automated cell production platform is used as a case study for validation. The results indicate that modifying an existing platform to support cleaning-in-place presents significant challenges. However, the article outlines general design guidelines for developing new biopharmaceutical production platforms that can accommodate automated cleaning.

## Introduction

In the biopharmaceutical and food industries, contamination is one of the highest risk factors for product quality and safety. In food production, (i) physical, (ii) chemical, or (iii) (micro)biological contamination of the product can lead to an unwanted or unacceptable taste or even poison the customer, which can lead to death and high costs for the healthcare systems (1, 2). These contaminants can originate from the food source, such as the raw food or animal, a processing step, or cross-contamination with other products or residues (3). In addition, thorough quality control of the food source and the production process, thorough cleaning of the production site is a major part of food safety and quality assurance.

In biopharmaceutical production, the importance of cleaning is at least as high. The consequences of contamination can be more severe, as these products are often directly injected or implanted into the patient. The risk of contamination lowering the efficacy of the product and injuring or killing the patient is very high (4, 5). Furthermore, in this production field, cross-contamination with cells from another patient is a major risk factor, along with contaminants from the outer environment or production personnel.

While in food production, many decontamination processes are already partly or completely automated (6), decontamination in the biopharmaceutical industry is still mainly performed manually and is therefore highly work-intensive and prone to human error and variation. Automation increases the efficiency, stability, and reproducibility of the process and allows for consistent data documentation, therefore leading to a consistently high product quality and enabling up-scaling of the process yield (7–9). In addition, the automation of decontamination processes reduces or eliminates the need for personnel to enter the production area, which could contaminate the area by distributing loose particles, viable organisms, or residues from a harmful biological product (10). In particular, it makes sense to also automate decontamination at a site that has fully automated production processes, as some of the required infrastructure is already available and it brings the site far closer to complete and fully self-sufficient automation.

The objective of this article was to investigate the feasibility of transferring technological approaches to the automatic decontamination of product-contact surfaces in food production to biopharmaceutical production. First, the regulations, requirements, and implementations in both industries are outlined, followed by the current decontamination technologies and concepts. Hereafter, possible automated decontamination applications in the biopharmaceutical industry are described. Finally, a sample automated cleaning platform for the biopharmaceutical industry is presented and analyzed in a dedicated cleaning simulation software.

## Regulatory requirements

To avoid contamination, several standards, guidelines, and regulations exist in both industries. The most important guideline that biopharmaceutical processes in the European Union must follow is good manufacturing practice (GMP). Even though it is only a guideline, most countries refer to it in their laws, making implementing the GMP guidelines mandatory. The GMP guidelines consist of several parts and annexes, of which Annex 1 is the most relevant regarding the production environment and cleaning (10). It describes the design requirements for a hygienic environment for processing, including decontamination and monitoring. Further important sources are two standards: ISO 14644, which also consists of several parts that describe requirements for cleanroom design, monitoring, and usage (11),; and ISO 17141, which contains recommendations for biocontamination control in cleanrooms (12). Both have many parallels with the GMP guideline.

In the food production industry, adherence to regulatory standards such as ISO 22000, GMP, and Hazard Analysis and Critical Control Points (HACCP) is crucial to ensure safety and quality. ISO 22000 is an international standard that specifies the requirements for a food safety management system (13). GMP guidelines are designed to ensure that food products are consistently produced and controlled according to quality standards, minimizing the risks involved in food production that cannot be eliminated by testing the final product (14). HACCP is a preventive approach that identifies potential hazards in production processes that can make food unsafe and designs measures to reduce these risks to a safe level (15). These standards collectively help to safeguard consumer health by ensuring comprehensive safety controls throughout the food supply chain.

## Current decontamination processes

Prior to disinfection, a thorough cleaning of the production area must be carried out in both food and biopharmaceutical applications. For a thorough cleaning, all unwanted matter, such as soil, food residues, dirt, grease, or any other objectionable matter, must be removed, as this matter can contaminate the product as well as provide attaching surfaces and protection for viable organisms. This step shall be henceforth referred to as *cleaning* and is expected to reduce the microorganism load by 99% (16). Second, any viable organisms need to be deactivated through *disinfection* or *sterilization.* Disinfection reduces the number of microorganisms on surfaces by 99.999% through the application of chemical agents or physical methods (16, 17). Sterilization is the process of killing or inactivating all microorganisms, including bacteria, viruses, and spores. The latter should be selected for GMP-compliant decontamination in biopharmaceutical production (18–20).

### Decontamination in the food industry

In the food industry, the common objective of cleaning procedures is to remove unwanted layers until the visual cleanliness of all surfaces is achieved (6). This includes removing various types of substances, such as carbohydrates, proteins, and fats, which require different cleaning agents and methods. For instance, alkaline cleaners are normally used for organic substances, while acidic cleaners are needed for inorganic substances (21, 22).

Cleaning procedures can comprise both manual cleaning and automatic cleaning-in-place (CIP) processes. The human factor provides flexibility and adaptivity. However, manual cleaning takes longer, the reproducibility of the cleaning results is often lower, and large equipment or tanks are not always accessible. Thus, to mitigate these drawbacks, CIP processes are commonly used.

Sinner's circle is often used to describe the balance between the most important factors that influence the cleaning process: (i) time, (ii) temperature, (iii) mechanical action, and (iv) chemical action. Moreover, the influence of the properties of the surface to be cleaned is considered by the extended Sinner’s circle (23–25).

CIP involves circulating cleaning solutions through pipes, tanks, and other equipment without dismantling them (6, 26–29). Cleaning processes in closed or immersed systems (e.g., pipes, heat exchangers, valves) are characterized by a two-phase system consisting of the surface to be cleaned and the cleaning fluid. Cleaning processes in open or non-immersed systems (e.g., filling machines, washing cabins, and tanks) are characterized by a three-phase system consisting of the surface to be cleaned, the cleaning liquid, and the atmosphere (27, 29–32). In open systems, the cleaning solutions are applied to the surface by means of static spray nozzles or spray balls, rotary spray cleaners, or rotary jet cleaners. The automation of these processes ensures sufficient cleaning and disinfection, reducing downtime and improving efficiency. CIP processes take place in a fixed sequence with defined rinsing, cleaning, and, if necessary, disinfection durations (29, 33)*.* Usually, the cleaning process is time-based, and current control systems are designed to fulfil the functional requirements of cleaning processes with fixed sequences. Through the underlying principles of cleaning validation and verification, commercial cleaning processes have been developed based on worst-case scenarios, resulting in excessive cleaning protocols (27, 34).

Chemical and physical methods are used for the sterilization and disinfection of food contact surfaces, such as packaging materials and machine components. In addition to conventional heat treatments using saturated steam or the application of common disinfectants such as hydrogen peroxide or peracetic acid, alternative decontamination methods, such as cold plasma, pulsed light, and UV-C irradiation, have gained increasing attention due to their effectiveness in decontaminating food contact surfaces at low temperatures and with reduced energy consumption (35, 36).

### Decontamination in the biopharmaceutical industry

The GMP guidelines define different sterility grades for biopharmaceutical production depending on the sensitivity of the processes performed in the corresponding area, with grade A the strictest, followed by grades B, C, and D (10). Cleaning methods such as blowing or ultrasound, which only loosen particles but do not remove them, or vacuum cleaning, which removes loose particles but not dirt or fluid sticking to the surface, are unsuitable for cleaning in grade A (37, 38). Therefore, the gold-standard method for manually cleaning environments for biopharmaceutical production is wiping. It requires specially designed sponges and tissues that emit sufficiently small amounts of particles (39). To ensure that all the particles are removed, the wiping pattern should be defined by wiping from top to bottom and in unidirectional tracks that overlap each other (40). This makes wiping very time-consuming and very challenging to automate.

The most established sterilizing method in the biopharmaceutical industry is vaporized hydrogen peroxide (H_2_O_2_). This gas is ventilated into the production area and is kept there for between 30 min and up to several hours before being removed (41). Another established sterilization method is heating. It is usually applied using autoclaves that kill contaminants with hot vaporized water (42).

Decontamination in biopharmaceutical production is currently mainly carried out manually. The usual sterilization methods in the biopharmaceutical industry are simple to automate, such as H_2_O_2_ vaporization, which only requires a ventilation pump system. Thus, the bottleneck for fully automated decontamination is cleaning, as only wiping and flushing with cleaning fluids are acceptable methods. Automating wiping in a biopharmaceutical production platform requires that a robot move a wiping tissue over every surface to clean. Furthermore, it needs to wipe in an organized pattern, normally from the center to the outside, to ensure that all the particles are removed from the sensitive areas. The robot either needs to be programmed to wipe every surface, which is challenging and prone to errors, or needs automated camera-assisted track planning to find all the surfaces by itself. Even in this scenario, it is difficult for a robot to reach all surfaces in the production area, as some may face away from the robot. A concept of a robotic system that allows for automated cleaning and sterilization by using flexible spray nozzles that apply cleaning and decontamination agents is presented by Haeusner et al. (43).

This article aims to evaluate the transferability of CIP concepts from food production using the example of a biopharmaceutical production platform (AUTOSTEM).

## The AUTOSTEM platform

The AUTOSTEM platform, which shall be used to discuss the application of CIP cleaning with spray nozzles in the biopharmaceutical industry, is a platform for automated bioreactor cultivation and the harvest of mesenchymal stromal cells (MSCs). As described in reference (9) and shown in Supplementary Figure 1, the platform consists of two compartments. The right compartment, which contains the bioreactors and freezing supplies and processes all the produced cells in closed systems, such as bioreactors, tubes, and flasks. As the cells have no contact with the air of the production site, this compartment’s cleanroom grade can be as low as EU GMP cleanroom grade D. In the left compartment, however, cells are transferred between flasks with a pipette and therefore are in contact with the air and require the highest cleanroom grade, grade A. To prevent air exchange between the compartments, they are separated by a wall that allows for object transfer through a hatch. Within the cleanroom grade A area, a vertically moving wall can further separate it temporarily to allow the opening of one part of the platform to refill supplies without the risk of contaminating the rest of the area. As the compartment with cleanroom grade A is in a far higher need of thorough cleaning, this part of the AUTOSTEM platform was chosen for the cleaning simulation.

## Simulation of spray cleaning in a biopharmaceutical production platform

Simulation is well-suited to designing CIP systems for maximum cleaning efficiency. For immersed components such as pipes and ducts, computational fluid dynamics-based methods (CFD) are especially effective but require expertise and are therefore not widely used despite their potential (44–46). In contrast, ADVISIM^3D^ is a very user-friendly commercial software for predicting cleaning in open systems, such as large vessels (47). It integrates computer-aided design (CAD) data of components or machinery, allowing users to add and place nozzles from a database within a familiar CAD environment. The software enables near real-time simulation of complex spray cleaning systems with multiple, even moving, nozzles and devices. Its simulation approach involves an experimental spray characteristics measurement of spraying devices, such as nozzles and static spray balls, in combination with an accurate projection onto the surfaces. Based on the fluid distribution (48), an integrated prediction model determines the cleaning effectiveness. This allows for virtual testing and optimization of cleaning systems, avoiding lengthy and costly iterative design and optimization experiments.

This method is applied here by simulating the cleaning of the AUTOSTEM cell production platform in ADVISIM^3D^. Typically, to allow for the CIP cleaning of large vessels, spraying devices are used that distribute cleaning fluid in a 360° arc, while rotating around up to two axes. Static spray balls (immobile metal balls with holes) are robust and reliable and are usually preferred in medical applications. Consequently, they are also used here to assess the challenges of using 360° cleaners in the AUTOSTEM platform. For this feasibility study, a spray shadow analysis was performed assuming a homogeneous fluid distribution over the full spraying angle.

As described above, the examined compartment of the AUTOSTEM can be split by a vertically moving wall that is not flush with the ceiling when retracted. All mobile installations were assumed to be removed for thorough external cleaning. The framework conditions were a goal of 100% coverage by direct jet impact, a minimum number of spraying devices, and the positioning of the devices in the ceiling area so as not to restrict the working area or chamber function, while reducing the likelihood of self-contamination. The results are shown in Supplementary Figure 2, with directly impacted areas in blue, untouched areas in gray, and the cross-sections of the cutting planes in red. The outcome of iterative manual optimization is shown in Supplementary Figure 2A, which shows two installed spray heads per chamber, and four in total.

Choosing suitable spray balls includes a consideration of their working range, i.e., the maximum distance that the spray balls can be from the surface so that it is still sufficiently cleaned by the fluid. A larger working range allows for fewer spraying devices. The illustrations show that the walls and ceilings were easily reached if the spray was unobstructed. Each installation, in general, must be sprayed by at least two, preferably three, opposing spray heads. In the AUTOSTEM platform, opening the connecting gate between the chambers during cleaning was beneficial and allowed the spray heads to reach the other chamber. The movement of the robot during the cleaning ensured complete coverage.

A very significant issue is illustrated in Supplementary Figure 2B, where large spray shadows are shown under the installations due to the spraying devices’ positioning close to the ceiling and the installations’ solid design. A potential solution could include designing permeable installations, making them removable, or using additional, adapted cleaning nozzles locally. Automated cleaning with a plausible number of spray heads was also very challenging when the installations were positioned near walls. This could be solved by positioning these further from walls, making the installations removable, or local clustering and cleaning with an additional spray head or nozzle. A third important issue is the cleaning of the hand glove passages, as shown in Supplementary Figure 2B on the right. While they are needed for manual interaction in the processes, their cleaning is not guaranteed with the given geometry. To promote their cleaning, an angled design, such as a conical shape, is advisable to allow fluid entry and drainage. Finally, there is a need for a drain in the floor plate, which must be at an incline to ensure complete fluid drainage. Recirculating the fluid during cleaning should be considered to increase efficiency.

## Conclusion

The intention of this article was to evaluate a concept for the automatic cleaning of an exemplary platform for automated biopharmaceutical production. While automatic solutions for sterilization are already available, cleaning in biopharmaceutical production is currently mainly conducted manually. Of all existing cleaning concepts, only wiping and flushing are appropriate for cleaning in highly sterile environments. While wiping is difficult and requires effort to automate, CIP cleaning by flushing is an already established cleaning method in the food industry. This method allows for thorough removal of particles and sterilization if the corresponding agents are added to the cleaning fluid. Once it is ensured that the spray nozzle installation can clean the required area completely, the method can be fully automated without the need for any human intervention. This reduces the risk of human contamination of the product and reduces the risk for employers coming into contact with potentially dangerous product residues.

The results of the simulation show that a cleaning process with spray nozzles is possible in the AUTOSTEM platform if certain redesigns are made. For instance, installations should be moved further from the walls or be designed to be removable for cleaning. In addition, the hand glove passage should be designed in a conical shape instead of a square shape. In general, to apply this cleaning method in a platform for biopharmaceutical production, the following should be implemented:

I Spray nozzles should only be mounted on the ceiling of the production area, as nozzles on the walls or on the bottom are difficult to clean or retain cleaning water. However, certain nozzle designs are able to be used on walls and the bottom if certain surfaces cannot be reached otherwise, such as nozzles with holes that allow for liquid to drain even when mounted horizontally or pointed upwards.
II All surfaces must be accessible to the cleaning fluid. This means that hidden or downfacing surfaces must be avoided, and surfaces should not be too close to one another or to the wall.
III The platform must be compliant with hygienic design criteria to ensure easy cleaning and to prevent the ingress, growth, and accumulation of hazards to product safety (49, 50). Sharp corners ≤90° must be avoided. Corners with angles smaller than 135° should have a minimum radius = 6 mm.
IV If possible, stainless steel or polymer materials should be used that are resistant to water and cleaning chemicals. Devices that are not resistant or are difficult to clean should be moved out of the area during cleaning.
V All surfaces must be self-drainable and should allow for a controlled flow of the cleaning fluid to an outlet. Since the equipment should be impermeable to microorganisms, the GMP guideline prohibits this outlet from being a drain (10). Therefore, a hatch should be installed that allows the draining of the used cleaning fluid into a sterilizable intermediate area. Once this area is filled completely or the cleaning process is finished, the area should then be sealed from the production area and open onto a drain to remove the fluid. The area should then be sterilized afterward. Alternatively, the fluid could be drained into a sterile disposable container that is replaced once full. Most existing platforms do not fulfill these requirements. Therefore, installing a CIP cleaning system in an existing platform is, in most cases, difficult or impossible, and redesigns would have to be made, as explained above. When designing a new platform with automated CIP cleaning, the described requirements should be considered from the very beginning of the development process. Finally, simulations, as demonstrated in this article, should be performed well before manufacturing and assembly to allow for changes and improvements where necessary.

## Data Availability

The original contributions presented in the study are included in the article/Supplementary Material, further inquiries can be directed to the corresponding author.
