# Supplementary material for: Feasibility analysis of automated cleaning in biopharmaceutical production using cleaning-in-place concepts from food production
Source: Front Med Technol. 2025 Sep 22;7:1540779. doi: 10.3389/fmedt.2025.1540779 (PMC12497818; doi:10.3389/fmedt.2025.1540779)
Supplement: Supplementary file 1 [file Datasheet1.docx]

Supplementary Material

Applying automated cleaning in biotechnological production by using methods from cleaning in food production

**Ferdinand Groten^1^, Chris Henze^2^, Matthias Jopppa^2^, Laura Herbst*^1^, Marc Mauermann^2^, and Robert H. Schmitt^1,3^**

^1^ Fraunhofer Institute for Production Technology IPT, Aachen, Germany

^2^ Fraunhofer Institute for Process Engineering and Packaging IVV, Dresden, Germany

3 Laboratory for Machine Tools and Production Engineering (WZL), RWTH Aachen University, Aachen, Germany

*** Correspondence:**Laura Herbst
laura.herbst@ipt.fraunhofer.de

# Supplementary Figures


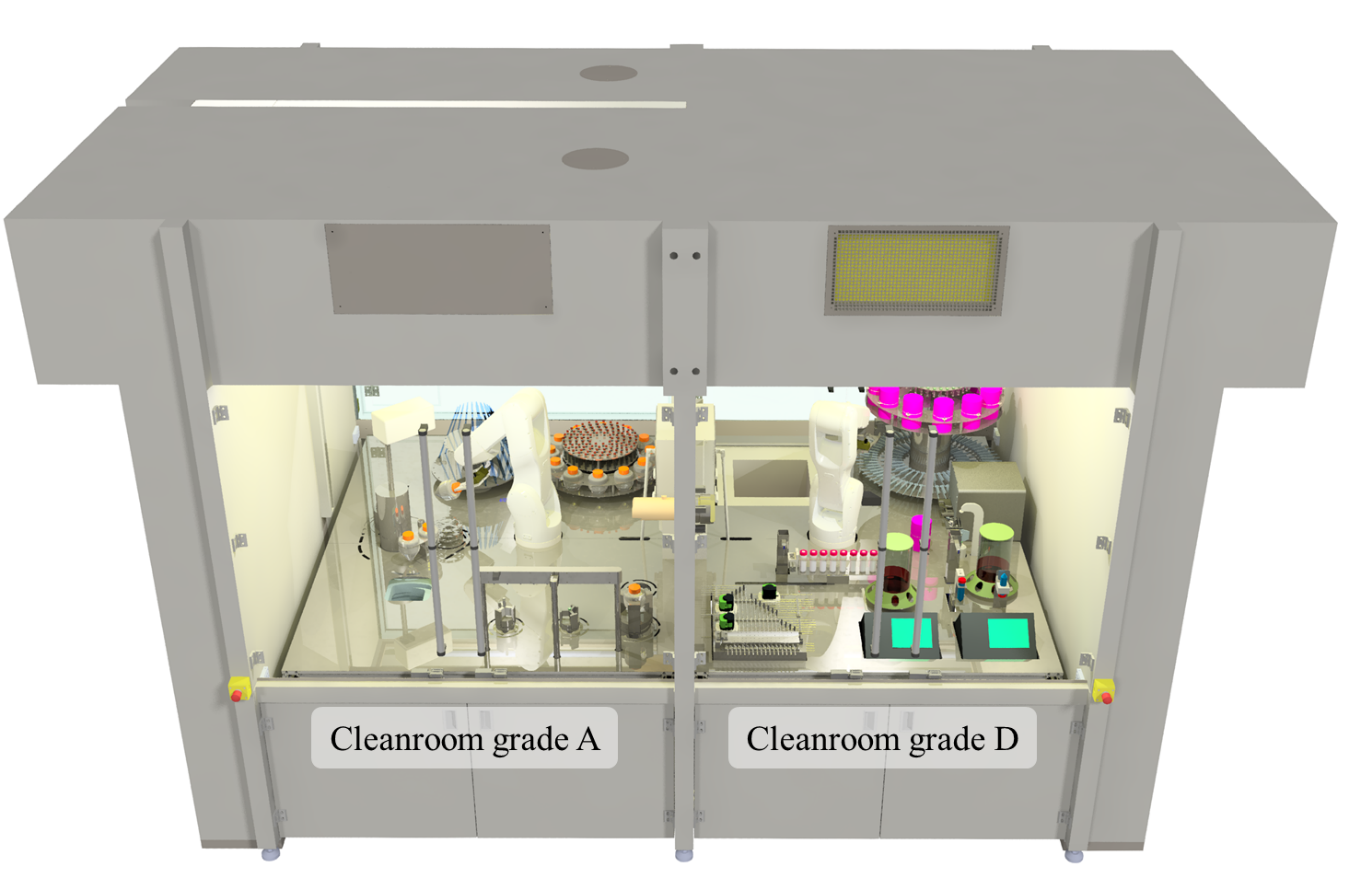


Figure 1: Rendered image of the AUTOSTEM platform with the lower cleanroom grade D on the right and the high cleanroom grade A on the left site, the latter of which is used for the evaluation.


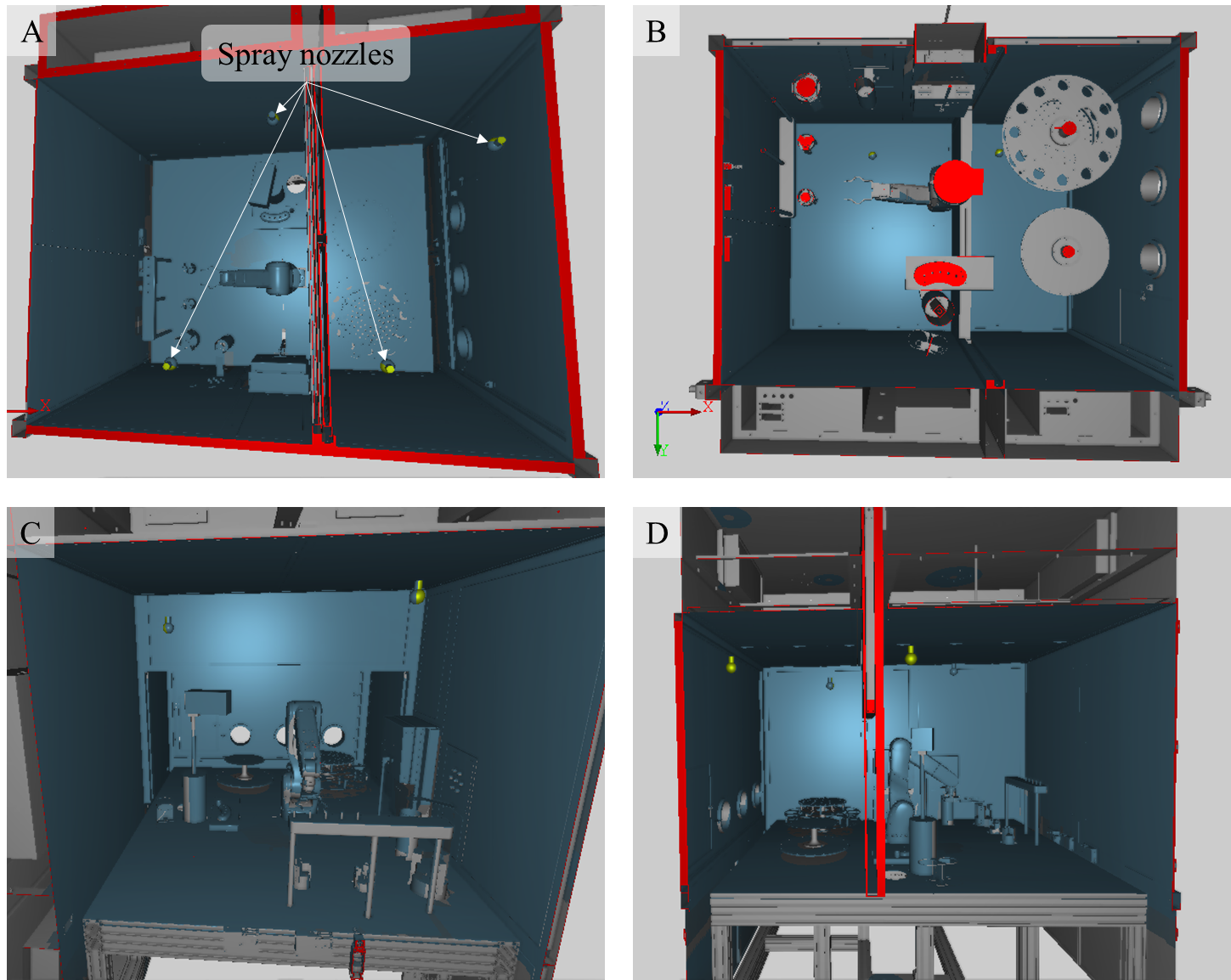


Figure 2: Simulation results of CIP cleaning in the AUTOSTEM platform in ADVISIM^3D^; top view (A), bottom view (B) and side views (C and D). Color code: light blue – direct impact of cleaning fluid, grey – spray shadow, not reached by cleaning fluid , red – cutting plane through solid parts, yellow – spray nozzles.
